# Automated Template-Based Hippocampal Segmentations from MRI: The Effects of 1.5T or 3T Field Strength on Accuracy

**DOI:** 10.1007/s12021-013-9217-y

**Published:** 2014-01-07

**Authors:** Kate E. Macdonald, Kelvin K. Leung, Jonathan W. Bartlett, Melanie Blair, Ian B. Malone, Josephine Barnes, Sebastien Ourselin, Nick C. Fox, for the Alzheimer’s Disease Neuroimaging Initiative

**Affiliations:** 1 Dementia Research Centre, UCL Institute of Neurology, Queen Square, London, WC1N 3BG UK; 2 Department of Medical Statistics, London School of Hygiene and Tropical Medicine, Keppel Street, London, WC1E 7HT UK; 3 Department of Medical Physics and Bioengineering, Centre for Medical Image Computing, 3rd Floor, Engineering Front Building, Malet Place, London, WC1E 6BT UK; 4 Dementia Research Centre, National Hospital for Neurology and Neurosurgery, Box 16, London, WC1N 3BG UK

**Keywords:** Hippocampus, Alzheimer’s disease, Magnetic resonance imaging, Automated segmentation, Field strength, Atrophy rate

## Abstract

Hippocampal volumetric measures may be useful for Alzheimer’s disease (AD) diagnosis and disease tracking; however, manual segmentation of the hippocampus is labour-intensive. Therefore, automated techniques are necessary for large studies and to make hippocampal measures feasible for clinical use. As large studies and clinical centres are moving from using 1.5 Tesla (T) scanners to higher field strengths it is important to assess whether specific image processing techniques can be used at these field strengths. This study investigated whether an automated hippocampal segmentation technique (HMAPS: hippocampal multi-atlas propagation and segmentation) and volume change measures (BSI: boundary shift integral) were as accurate at 3T as at 1.5T. Eighteen Alzheimer’s disease patients and 18 controls with 1.5T and 3T scans at baseline and 12-month follow-up were used from the Alzheimer’s Disease Neuroimaging Initiative cohort. Baseline scans were segmented manually and using HMAPS and their similarity was measured by the Jaccard index. BSIs were calculated for serial image pairs. We calculated pair-wise differences between manual and HMAPS rates at 1.5T and 3T and compared the SD of these differences at each field strength. The difference in mean Jaccards (manual and HMAPS) between 1.5T and 3T was small with narrow confidence intervals (CIs) and did not appear to be segmentor dependent. The SDs of the difference between volumes from manual and automated segmentations were similar at 1.5T and 3T, with a relatively narrow CI for their ratios. The SDs of the difference between BSIs from manual and automated segmentations were also similar at 1.5T and 3T but with a wider CI for their ratios. This study supports the use of our automated hippocampal voluming methods, developed using 1.5T images, with 3T images.

## Introduction

The hippocampus is one of the earliest site of pathological changes (Braak and Braak 1991) and atrophy (Jack et al. 1999; Ridha et al. 2006) in Alzheimer’s disease (AD). Magnetic resonance imaging (MRI) based measurement of volume and volume change in the hippocampus may be useful markers for AD diagnosis and tracking progression (Dubois et al. 2010; Henneman et al. 2009; Jack et al. 2005, 2008b; Wang et al. 2009). However, the “gold standard” measurement technique of manual segmentation is very labour intensive, taking up to 45 min per side, and is impractical for large studies or clinical trials, making the use of automated segmentation techniques necessary. Hippocampal Multi-atlas Propagation and Segmentation (HMAPS) (Leung et al. 2010a) is a template-based automated hippocampal segmentation technique that uses a library of hippocampal regions that were segmented on a set of 1.5 Tesla (T) MR scans. HMAPS has been shown to be accurate on 1.5T scans, but using the same template library on scans acquired at a different field strength may yield different results. Despite higher field strengths having the benefit of an improved signal to noise ratio, artefacts such as susceptibility and chemical shift can be more apparent and more important at higher field strengths (Bernstein et al. 2006; Farahani et al. 1990; Parizel et al. 1994). Additionally, measurement of volumes of different brain areas may be affected by changes in field strength. For example, Kruggel et al (2010) showed that grey matter and white matter volumes calculated from a tissue classification algorithm can vary within subject across different field strengths. Also, preliminary results from Chow et al (2013) suggest that 3T images may be able to detect volume differences that are not apparent at 1.5T. In contrast, others have found no significant differences between manually segmented regions (hippocampus and amygdala) on 1.5T and 3T images (Briellmann et al. 2001; Scorzin et al. 2008) and Ho et al (2010) found that sample size estimates for detecting a slowing of mild cognitive impairment using Tensor Based Morphology did not differ between 1.5T and 3T scans.

Furthermore, automated atrophy rate measurements that rely on measuring intensities within the scan, such as the boundary shift integral (BSI), may also be affected by changes in field strength due to an increase in contrast to noise ratio (Hart et al. 1983). Because higher field strengths are becoming more commonly used clinically and in therapeutic trials, it is important to ensure that automated measures such as HMAPS are generalisable to higher field strengths. The aim of this report was to investigate whether HMAPS is as accurate on 3T scans as on 1.5T scans and whether volume change estimated using BSI combined with these automated segmentations was similar using 1.5T and 3T serial image pairs. We also investigated the ability of HMAPS volumes and atrophy rates to distinguish between control subjects and subjects with AD and whether the predictive ability differed between 1.5T and 3T scans.

## Methods

### Subjects and Scans

Subjects included were AD subjects and controls from the Alzheimer’s Disease Neuroimaging Initiative (ADNI) cohort who had a 1.5T and 3T scan at both baseline and 12 months. There were 28 AD and 50 control subjects meeting these criteria. ADNI is a multi-centre public/private funded longitudinal study investigating adult subjects with AD, amnestic MCI, and normal cognition. Participants underwent baseline and periodic clinical and neuropsychometric assessments and serial MRI. Details are available at http://www.adni-info.org. Written informed consent was obtained, as approved by the Institutional Review Board at each of the participating centres. ADNI inclusion and exclusion criteria are detailed elsewhere (http://www.adni-info.org/Scientists/Pdfs/ADNI_Protocol_Extension_A2_091908.pdf).

All scans were downloaded from http://www.loni.ucla.edu/ADNI. These scans were pre-processed by the ADNI researchers. The ADNI T1 images were corrected for distortion due to gradient non-linearity (Jovicich et al. 2006) (using N3 (Sled et al. 1998) for all images and B1 (Narayana et al. 1988) where required), and scaling-corrected based on phantom measures (Jack et al. 2008a).

For the ADNI study, 1.5T scans and 3T scans of the same time-point were not always conducted on the same day and therefore the scan intervals sometimes differed for 1.5T and 3T scans pairs. Because differences in scan intervals may have implications for measurement error, we minimised this effect by excluding potential subjects with scan interval differences of more than 60 days between 1.5T and 3T scan pairs. We excluded three AD and six control subjects that had scan interval differences of more than 60 days. Scan pairs were visually assessed and subjects with poor quality scan pairs were excluded in this study. There were 18 remaining AD subjects and 26 control subjects with good quality scan pairs. We selected 18 of the good quality control subjects to give equal numbers of AD and control subjects.

As part of the manual segmentation protocol, we rigidly registered the 1.5T scans to the MNI305 atlas. In order to allow the direct comparison of the segmentations between 1.5T and 3T scans, we rigidly registered the 3T scans to the resliced 1.5T scans in the MNI305 space. The left hippocampus of all baseline scans (1.5T and 3T) was segmented automatically using HMAPS and manually by two trained segmentors (segmentor 1 (S1) and segmentor 2 (S2)) independently using an adapted version of a protocol originally described by Watson et al. (1992) (see appendix for details of the manual protocol). The segmentors were blinded to field strength and patient diagnosis, and neither segmented any of the hippocampi in the template library.

Prior to calculating the BSI, scan pairs underwent differential bias correction (DBC) to correct for inhomogeneity differences between the scan pairs (Lewis and Fox 2004). The DBC was performed because varying amounts of intensity inhomogeneity were still present in the pre-processed scans after N3 correction. Because BSI directly compares the intensity between the baseline and repeat images, DBC is applied to correct the bias between the two images to improve its robustness to the intensity inhomogeneity artifacts. The baseline regions were used to calculate atrophy rates using the BSI with a double window approach, in order to capture changes across both the CSF-hippocampal border and the white matter-hippocampal border (Leung et al. 2010a, b). This provided an automated HMAPS-BSI measure at 1.5T and 3T and a corresponding semi-automated atrophy rate measure S1-BSI and S2-BSI for each manual segmentor.

### Statistics

We used the Jaccard index as a measure of similarity, which is defined as the magnitude of the intersection divided by the magnitude of the union of two regions. Jaccard indices were calculated for the baseline segmentations to assess the accuracy of the HMAPS volumes. We calculated the pair-wise difference between the Jaccard of S1 and HMAPS at 1.5T and the Jaccard of S1 and HMAPS at 3T. A one sample t-test was used to assess whether the mean of these differences was significantly different to zero, which would indicate systematically better agreement with manual for 1.5T compared to 3T (or vice-versa). Pair-wise differences were also calculated and assessed in the same way between the Jaccard indices comparing S2 and HMAPS and comparing S1 and S2 across field strengths to assess if there was a segmentor bias. Intraclass correlations (ICC) were estimated on baseline manual segmentations by S1 and S2 using the loneway command in Stata (version 12).

To assess bias in baseline volumes we performed paired t-tests (separately for 1.5T and 3T) comparing HMAPS volume and the mean of S1 and S2 volumes (S1-S2-mean-volume). We calculated the SD of pair-wise differences between S1-S2-mean-volume and HMAPS volume separately for 1.5T and 3T to evaluate agreement between manual and automated volumes at different field strengths. We calculated the ratio of these SDs to quantify the difference in agreement between 1.5T and 3T volumes. Bias-corrected and accelerated 95 % bootstrap confidence intervals (10,000 bootstrap replicates) were calculated for the SDs and their ratio.

Annualised atrophy rate as a percentage of baseline volume was calculated using the BSIs on a log scale and back-transformed. To assess bias in atrophy rates we performed t-tests (separately for 1.5T and 3T) comparing HMAPS atrophy rates to the mean of rates from S1 and S2 (S1-S2-mean-BSI). Separately for 1.5T and 3T, we calculated the SD of pair-wise differences between S1-S2-mean-BSI and HMAPS-BSI as a measure of agreement between atrophy rates derived from manual and HMAPS segmentations. We also calculated the ratio of these two SDs to quantify the difference in agreement between 1.5T and 3T atrophy rates. Bias-corrected and accelerated 95 % bootstrap confidence intervals (10,000 bootstrap replicates) were calculated for the SDs and their ratio.

To quantify the ability of a) HMAPS volumes and b) hippocampal BSI atrophy rates to discriminate between AD subjects and controls, we estimated the area under the receiver operating characteristic curve (AUC) for each measure and used the Wilcoxon rank-sum test to test the null hypothesis that the AUC equalled 0.5 (which corresponds to no predictive value). We calculated 95 % confidence intervals for the AUCs and compared AUCs between measures using the Stata commands roccomp and comproc.

### Post Hoc Analysis

Annualised atrophy rate as mm^3^ loss per year was calculated using the BSI. To assess bias in atrophy rates we performed t-tests (separately for 1.5T and 3T) comparing HMAPS atrophy rates to the mean of rates from S1 and S2 (S1-S2-mean-BSI).

## Results

Baseline characteristics are shown in Table 1. The AD subjects included more APOE ε4 carriers and had lower MMSE scores than controls, as expected.

**Table 1:** Baseline characteristics for controls and AD subjects. Values represent mean (standard deviation [SD]) except for gender and APOE ε4 carrier status for which values are number (percent)

	AD (*n* = 18)	Control (*n* = 18)
Age at baseline 1.5T scan (years)	74.7 (8.1)	74.4 (4.9)
Age at baseline 3T scan (years)	74.8 (8.1)	74.5 (4.9)
1.5T scan interval (years)	1.05 (0.05)	1.07 (0.05)
3T scan interval (years)	1.01 (0.06)	1.01 (0.04)
Male	6 (33.3)	7 (38.9)
APOE ε4 carrier	11 (61.1)	7 (38.9)
MMSE (score out of 30)	23.0 (2.1)	29.4 (0.7)

### Jaccard Indices

Means and SDs of Jaccard indices are shown in Table 2.

**Table 2:** Mean (SD) Jaccard indices segmentor 1 (S1), segmentor 2 (S2) and HMAPS for the left hippocampus

	AD (*n* = 18)	Controls (*n* = 18)	All subjects (*n* = 36)
Jaccard indices comparing HMAPS and manual			
S1 and HMAPS at 1.5T	0.806 (0.025)	0.813 (0.025)	0.810 (0.025)
S1 and HMAPS at 3T	0.817 (0.019)	0.808 (0.025)	0.813 (0.022)
S2 and HMAPS at 1.5T	0.802 (0.024)	0.812 (0.020)	0.807 (0.022)
S2 and HMAPS at 3T	0.801 (0.022)	0.798 (0.024)	0.800 (0.023)
HMAPS at 1.5T and 3T	0.825 (0.034)	0.842 (0.028)	0.833 (0.032)
Inter- and intra-segmentor Jaccard Indices			
S1 and S2 at 1.5T	0.860 (0.026)	0.870 (0.029)	0.865 (0.028)
S1 and S2 at 3T	0.875 (0.029)	0.875 (0.025)	0.875 (0.027)
S1 at 1.5T and 3T	0.851 (0.030)	0.870 (0.023)	0.861 (0.028)

The difference in mean Jaccards comparing S1 and HMAPS between 1.5T and 3T scans was small (0.003 [95 % CI: -0.008, 0.014], *p* = 0.56) with higher Jaccards at 3T. The difference in mean Jaccards comparing S2 and HMAPS between 1.5T and 3T was also small with a narrow confidence interval (0.007 [95 % CI: -0.002, 0.017], *p* = 0.56) with higher Jaccards at 1.5T. There was evidence that the difference in mean Jaccards comparing S1 and S2 was higher for 3T than 1.5T scans (0.010 [95 % CI: 0.001, 0.019], *p* = 0.02).

The inter-rater ICC (in this sample of 18 AD and 18 controls) for S1 and S2 was 0.971 (95 % CI: 0.952, 0.990) on 1.5T scans and 0.980 (95 % CI: 0.967, 0.993) on 3T scans.

### Volumes

The means and SDs of baseline volumes are shown in Table 3.

**Table 3:** Mean (SD) baseline hippocampal volumes (left side only) for segmentor 1 (S1), segmentor 2 (S2) and HMAPS at 1.5T and 3T

	AD (*n* = 18)	Controls (*n* = 18)	All subjects (*n* = 36)
Baseline volumes (ml) at 1.5T			
S1 at 1.5T	2.01 (0.53)	2.68 (0.46)	2.34 (0.60)
S2 at 1.5T	2.06 (0.58)	2.74 (0.47)	2.40 (0.63)
HMAPS at 1.5T	1.95 (0.55)	2.63 (0.42)	2.23 (0.54)
Baseline volumes (ml) at 3T			
S1 at 3T	2.00 (0.46)	2.71 (0.47)	2.36 (0.58)
S2 at 3T	2.01 (0.49)	2.69 (0.45)	2.35 (0.58)
HMAPS at 3T	1.91 (0.46)	2.54 (0.43)	2.23 (0.54)

Across all subjects, there was some evidence of a difference in volumes of S1 and S2 for 1.5T (mean difference 0.06 ml [95 % CI 0.012, 0.10], *p* = 0.015) with higher volumes for S2. There was evidence that the HMAPS-volume was on average 0.08 ml [95 % CI 0.03, 0.13, *p* = 0.002] lower than S1-S2-mean-volume at 1.5T. Mean volumes for S1 and S2 were similar at 3T (mean difference 0.002 ml [95 % CI -0.04, 0.04], *p* = 0.92). For 3T there was evidence that HMAPS volumes were lower than S1-S2-mean-volume (mean difference 0.13 ml [95 % CI 0.08, 0.18], *p* < 0.001).

The SD of the difference between S1-S2-mean-volume and HMAPS-volume was similar for 1.5T scans (0.15 ml [95 % CI 0.13, 0.18]) and 3T scans (0.15 ml [95 % CI 0.12, 0.19]), suggesting similar levels of agreement. The ratio of these SDs was 0.99 [95 % CI 0.85, 1.22]. The relatively narrow confidence interval for the ratio indicates moderate evidence that agreement between manual and HMAPS volumes is similar at 1.5T and 3T.

### Atrophy Rates

Table 4 shows the means and SDs for atrophy rates at 1.5T and 3T.

**Table 4:** Mean (SD) hippocampal atrophy rates for segmentor 1 (S1), segmentor 2 (S2) and HMAPS at 1.5T and 3T for the left side only

	AD (*n* = 18)	Control (*n* = 18)	All subjects (*n* = 36)
BSI (%/year) rates at 1.5T			
S1 at 1.5T	3.85 (2.24)	0.08 (1.75)	1.97 (2.75)
S2 at 1.5T	3.46 (2.14)	−0.10 (1.77)	1.68 (2.65)
HMAPS at 1.5T	4.03 (2.50)	0.06 (1.79)	2.05 (2.94)
BSI (%/year) rates at 3T			
S1 at 3T	3.42 (2.00)	0.42 (1.86)	1.92 (2.44)
S2 at 3T	3.22 (1.92)	0.31 (1.89)	1.77 (2.39)
HMAPS at 3T	3.63 (2.49)	0.43 (1.92)	2.03 (2.73)

Across all subjects, there was evidence that atrophy rates from S2 were smaller than for S1 for 1.5T (mean difference 0.29 % points [95 % CI: 0.10, 0.47], *p* = 0.003). There was borderline evidence of a difference between S1-S2-mean-BSI and HMAPS-BSI for 1.5T with HMAPS rates being higher (mean difference 0.22 % points [95 % CI -0.01, 0.45], *p* = 0.06). There was evidence that atrophy rates from S2 were smaller than for S1 for 3T (mean difference 0.15 % points [95 % CI 0.03, 0.27], *p* = 0.01). For 3T the difference between S1-S2-mean-BSI and HMAPS rates was of a similar magnitude and in the same direction as for 1.5T (mean difference 0.18 % points [95 % CI -0.04, 0.40], *p* = 0.10). The CI of this difference was also similar to the CI for 1.5T atrophy rates.

The SD of the difference between S1-S2-mean-BSI and HMAPS-BSI was similar for 1.5T scans (0.68 % points [95 % CI 0.54, 0.89]) and 3T scans (0.65 % points [95 % CI 0.47, 0.96]), suggesting similar levels of agreement. The ratio of these SDs was 1.04 (95 % CI 0.59, 1.41), with the wider CI indicating that we have less confidence that is in truth similar at 1.5T and 3T.

Because HMAPS rates were higher than manual rates in 1.5T and 3T and manual volumes were found to be larger than HMAPS volumes, we repeated the atrophy rate analyses above using mm^3^ loss per year as the atrophy rate measure rather than percentage of baseline volume to lessen the influence of volume from the atrophy rate measure. These analyses are presented in the Post hoc analysis section below.

### Post Hoc Analysis

Table 5 shows the means and SDs for atrophy rates (in mm^3^ loss per year) at 1.5T and 3T.

**Table 5:** Mean (SD) hippocampal atrophy rates (mm^3^ loss per year) for segmentor 1 (S1), segmentor 2 (S2) and HMAPS at 1.5T and 3T for the left side only

	AD (*n* = 18)	Controls (*n* = 18)	All subjects (*n* = 36)
BSI (mm^3^ loss per year) rates at 1.5T			
S1 at 1.5T	76.18 (43.68)	0.03 (45.80)	38.10 (58.62)
S2 at 1.5T	70.64 (42.22)	-4.33 (47.33)	33.15 (58.22)
HMAPS at 1.5T	75.68 (41.94)	1.01 (42.52)	38.35 (56.27)
BSI (mm^3^ loss per year) rates at 3T			
S1 at 3T	65.07 (38.29)	11.57 (47.96)	38.32 (50.65)
S2 at 3T	61.82 (38.02)	7.56 (47.51)	34.69 (50.55)
HMAPS at 3T	66.04 (45.25)	10.22 (45.89)	38.13 (53.09)

Across all subjects, there was evidence that atrophy rates from S2 were smaller than for S1 for 1.5T (mean difference 4.95 mm^3^ [95 % CI: 1.87, 8.03], *p* = 0.0025). The S1-S2-mean-BSI was similar to HMAPS-BSI for 1.5T (mean difference 2.72 mm^3^ [95 % CI -1.36, 6.80], *p* = 0.18). There was evidence that atrophy rates from S2 were smaller than for S1 for 3T (mean difference 3.64 mm^3^ [95 % CI 0.64, 6.64], *p* = 0.019). For 3T the difference between S1-S2-mean-BSI and HMAPS rates was small (mean difference 1.62 mm^3^ [95 % CI -2.40, 5.64], *p* = 0.42).

The SD of the difference between S1-S2-mean-BSI and HMAPS-BSI was similar for 1.5T scans (12.05 mm^3^ [95 % CI 9.69, 15.58]) and 3T scans (11.89 mm^3^ [95 % CI 8.83, 17.38]), suggesting similar levels of agreement. The ratio of these SDs was 1.01 (95 % CI 0.64, 1.41), with the wide CI indicating again that we have less confidence that, in truth, agreement between manual BSI rates and HMAPS BSI is similar at 1.5T and 3T.

### Group Separation

Table 6 shows AUCs, 95 % confidence intervals, and *p*-values (testing the null hypothesis of no predictive value) for all BSI and baseline HMAPS volumes.

**Table 6:** Area under the receiver operator curve (AUC) representing the value of 12-month atrophy rate (% baseline volume) and baseline volumes in discriminating between controls and AD subjects, 95 % confidence intervals, and *p*-values testing the null hypothesis that the AUC equals 0.5 for each of the measures

	AUC	95 % confidence interval		*p*-value
		Lower	Upper	
1.5T HMAPS volume	0.833	0.694	0.973	0.0006
3T HMAPS volume	0.836	0.703	0.970	0.0006
1.5T HMAPS BSI	0.917	0.825	1.000	<0.0001
3T HMAPS BSI	0.861	0.742	0.980	0.0002

Both 1.5T and 3T measures of atrophy rate and baseline volume had high discriminative ability, although confidence intervals for the AUC were wide (controls vs. ADs). Discriminative ability was very similar using 1.5T and 3T volumes (AUC difference 0.003 [95 % CI -0.03, 0.06]). Discriminative ability was also very similar using 1.5T and 3T atrophy rates (AUC difference 0.06 [95 % CI -0.06, 0.19]).

## Discussion

The difference in mean Jaccard index comparing the first manual segmentor (S1) and HMAPS between 1.5T and 3T scans was small, suggesting that HMAPS is similarly accurate at generating volumes at both field strengths. Likewise, the variation of the difference between manual volumes and HMAPS volumes was similar on 1.5T and 3T scans, with a relatively narrow CI for the ratio of SDs indicating a moderate level of confidence that agreement is similar. Furthermore, the differences between mean Jaccard index comparing a second manual segmentor (S2) and HMAPS between 1.5T and 3T scans were also small, suggesting that the accuracy of HMAPS is not segmentor dependent. Our results are consistent with Lötjönen et al. (2011) who examined differences in hippocampal segmentations using an automated multi-atlas segmentation method on 1.5T and 3T images and found a high ICC of 0.98 between 1.5T and 3T segmentations. Our data gave a comparable ICC of 0.97 between 1.5T and 3T HMAPS segmentations.

Agreement between the volumes generated by S1 and S2 was slightly better at 3T than at 1.5T. Similarly, we found evidence that S1 volumes were significantly lower than S2 volumes at 1.5T, but not at 3T. These findings may be explained by the observation that the grey matter and white matter boundaries were visually clearer on the 3T scans, which would make manual delineation easier and more consistent. Likewise, Briellmann et al. (2001) found that manual measurement error was slightly lower on 3T compared with 1.5T scans.

We found evidence that HMAPS volumes were smaller than manual volumes at 1.5T and 3T. Leung et al. (2010a) report smaller automated volumes relative to manual segmentation with overall mean differences (for control, MCI and AD subjects, on 1.5T images from ADNI) of 27 mm^3^ and 101 mm^3^ for two separate groups of subjects. This is comparable to the mean difference of 0.08 ml (80 mm^3^) for the 1.5T images in this paper.

We found borderline evidence that HMAPS atrophy rates (% per year) differed from rates derived from manual segmentors at 1.5T but no evidence of a difference at 3T. The difference between HMAPS atrophy rates and manual segmentations at 1.5T may be driven by the lower atrophy rates of S2. The lower atrophy rates of S2 compared with S1 and HMAPS could be due to the tendency of S2 for larger volumes, which affects our atrophy rate measure as baseline volume serves as a denominator for this measure. Consequently we repeated this analysis using mm^3^ loss per year and found no evidence of a difference between HMAPS and manually derived rates at 1.5T and 3T. The variation of the difference between manually derived rates and HMAPS atrophy rates was similar for 1.5T scans and 3T scans (using both % per year and mm^3^ loss per year), suggesting that agreement between atrophy rates derived from the automated procedure with BSI and from manual procedure with BSI is similar on 3T and 1.5T scans. However, the large confidence interval for the ratio of SDs indicates that there is a large amount of uncertainty about this finding. The implication of this uncertainty may mean that if longitudinal studies include scans across subjects at 1.5T and 3T it would be prudent to take this into account in the analyses.

Finally, we found that the ability of HMAPS volumes and atrophy rates to distinguish between control subjects and subjects with AD was high, with AUC values between 0.83 and 0.92, with a similar level of predictive ability at 1.5T and 3T for both volumes and atrophy rates.

Strengths of this study included the multi-site nature of the study, and having multiple field strengths at same time-points, which allowed for comparison of field strengths. Limitations included the small number of subjects, which limits the precision with which differences in agreement between 1.5T and 3T can be estimated. Also, 1.5T and 3T scans for the same time-points were not always conducted on the same day, meaning that scan intervals differed between scan pairs which may affect measurement error. This effect was minimised by selecting subjects with scan intervals that were less than 60 days different between 1.5T and 3T scans. Another limitation is that the ADNI researchers designed the MRI protocol such that the tissue contrast of the 3T scans closely matched the 1.5T scans (Ho et al. 2010). This design may have contributed to the similarities we found between 1.5T and 3T. It is also important to note that this paper only examined differences in field strength. Differences between hippocampal volumes and atrophy rates could also be due to other factors that were not examined in this investigation such as scanner manufacturer or head coil. A further consideration is that it is not known whether the performance of the ADNI image pre-processing corrections is similar at different field strengths.

The results reported in this paper relate to a 1.5T hippocampal template library and its application to 1.5T and 3T image pairs. A template library created at 1.5T will not benefit from the improved contrast seen at 3T and as such the results of applying a 3T-based template library may differ to those presented here. Further, we investigated AD-control differences as part of this study, however, techniques such as HMAPS and associated BSIs may be most useful in detecting pre-clinical AD. As such the application of HMAPS and BSI to an MCI group to assess the sensitivity to conversion to AD and whether this is influenced by field strength would be useful.

## Conclusion

We found a high level of agreement between volumes generated by an automated segmentation technique, HMAPS, relative to manual measurements on MR scans acquired at 1.5T and 3T. Further, when generating atrophy rates for both the HMAPS and manual baseline regions using an automated technique for quantifying volume loss (BSI), agreement between HMAPS and manual atrophy rate measures was similar for 1.5T and 3T scans. However, for both volumes and atrophy rates, the CIs for the difference in agreement between 1.5T and 3T indicated that, in truth, differences in agreement may be larger. HMAPS volumes and atrophy rates discriminated well between controls and AD subjects, and there was no evidence of a difference in predictive ability between 1.5T and 3T. This study supports the use of HMAPS automated hippocampal measures, developed using 1.5T images, with 3T images.

## Information Sharing Statement

Data used in preparation of this article were obtained from the Alzheimer’s Disease Neuroimaging Initiative (ADNI) database (adni.loni.ucla.edu). As such the investigators within the ADNI contributed to the design and implementation of ADNI and/or provided data but did not participate in analysis or writing of this report. A complete listing of ADNI investigators can be found at: http://adni.loni.ucla.edu/wp-content/uploads/how_to_apply/ADNI_Acknowledgement_List.pdf.

All images used in this manuscript are freely available to the public. These data are available from the website cited in the Methods section.

## Appendix

We defined the hippocampus as including the dentate gyrus, the hippocampus proper, the subiculum and the alveus. Measurements were taken from every coronal slice from the posterior to anterior boundaries using a standard neuroanatomical atlas (Duvernoy 1998). The posterior boundary of the hippocampus was defined as the coronal slice where the longest length of the crus of the fornix was seen (Watson et al. 1992). The hippocampus was bounded superiorly, medially and laterally by CSF and inferiorly by the white matter of the subiculum. The hippocampal head was delineated from the amygdala by inclusion of the alveus, best seen as high signal intensity band on sagittal sections. We applied a minimum threshold of 70 % of mean brain intensity to improve consistency by excluding voxels which were predominantly cerebrospinal fluid.

## Abbreviations

HMAPS: Hippocampal multi-atlas propagation and segmentation
BSI: Boundary shift integral
ADNI: Alzheimer’s disease neuroimaging initiative
S1: Segmentor 1
S2: Segmentor 2
SD: Standard deviation
